# Supplementary material for: Specificity and mechanism of the double-stranded RNA-specific J2 monoclonal antibody
Source: bioRxiv. 2025 May 10:2025.05.09.649859. Preprint. [Version 1] doi: 10.1101/2025.05.09.649859 (PMC12190177; doi:10.1101/2025.05.09.649859)
Supplement: Supplement 1 [file media-1.pdf]

Supplementary Materials for:

Specificity and mechanism of the double-stranded RNA-specific J2 monoclonal antibody

Charles Bou-Nader, Kevin M. Juma, Ankur Bothra, Andrew J. Brasington, Rodolfo Ghirlando, Motoshi Suzuki, David N. Garboczi, Stephen H. Leppla, and Jinwei Zhang

Supplementary Tables 1-2
Supplementary Figures 1-10

Ligand (J2 IgG)	Analyte (dsRNA)	k_{on} (×10^5 $M^{-1}s^{-1}$)	k_{off} (×10^{-3} s^{-1})	K_d (nM, kinetics)	K_d (nM, steady state)
WT	6-bp	N.D.	N.D.	N.D.	N.D.
WT	10-bp	N.D.	N.D.	N.D.	N.D.
WT	14-bp	2.3 ± 1.9	220.3 ± 27.8	1400 ± 800	2100 ± 600
WT	20-bp	5.9 ± 2.8	72.1 ± 2.5	150 ± 80	120 ± 10
WT	30-bp	3.5 ± 5.5	10.3 ± 15.3	39 ± 10	12 ± 28
WT	40-bp	5.3 ± 0.2	17.2 ± 2.8	33 ± 6	80 ± 15
WT	50-bp	N.D.	N.D.	N.D.	22 ± 8
HC A30P	20-bp	2.1 ± 1.9	55.1 ± 7. 0	28 ± 14	120 ± 20
HC Y50A	20-bp	N.D.	N.D.	N.D.	N.D.
HC Y50F	20-bp	15. ± 5.3	124.7 ± 29.1	94 ± 49	430 ± 40
HC Y52A	20-bp	N.D.	N.D.	N.D.	N.D.
HC Y52F	20-bp	5.8 ± 0.3	122.7 ± 7.1	210 ± 20	220 ± 0
HC Y54A	20-bp	5.1 ± 0.2	138.0 ± 7.8	270 ± 10	300 ± 30
HC Y54F	20-bp	5.7 ± 0.8	140.3 ± 6.8	250 ± 40	710 ± 170
HC N55A	20-bp	N.D.	N.D.	N.D.	N.D.
HC N101A	20-bp	N.D.	N.D.	N.D.	N.D.
HC N101R	20-bp	N.D.	N.D.	N.D.	N.D.
HC P102A	20-bp	N.D.	N.D.	N.D.	N.D.
HC W104A	20-bp	N.D.	N.D.	N.D.	N.D.
LC Y31A	20-bp	8.0 ± 3.3	136.8 ± 91.4	207 ± 21	940 ± 560
LC Y31F	20-bp	7.6 ± 1.0	97.0 ± 3.5	130 ± 20	360 ± 60
LC S33R	20-bp	N.D.	N.D.	N.D.	N.D.
LC Y38F	20-bp	9.5 ± 0.1	79.0 ± 13.4	83 ± 14	150 ± 20
LC W56A	20-bp	N.D.	N.D.	N.D.	N.D.
LC Y101A	20-bp	N.D.	N.D.	N.D.	N.D.
WT	GC_0%, 30 bp	5.2 ± 1.9	146.7 ± 4.0	320 ± 140	290 ± 60
WT	GC_3%, 30 bp	1.2 ± 1.7	19.1 ± 24.8	190 ± 50	220 ± 90
WT	GC_17%, 30 bp	2.4 ± 0.2	16.4 ± 0.8	470 ± 30	36 ± 22
WT	GC_33%, 30 bp	1.4 ± 0.2	148.3 ± 6.7	1100 ± 200	460 ± 90
WT	GC_47%, 30 bp	1.6 ± 1.3	19.5 ± 2.9	560 ± 810	110 ± 20
WT	GC_63%, 30 bp	N.D.	N.D.	N.D.	N.D.
WT	GC_77%, 30 bp	N.D.	N.D.	N.D.	N.D.
WT	GC_90%, 30 bp	N.D.	N.D.	N.D.	N.D.
WT	GC_100%, 30 bp	N.D.	N.D.	N.D.	N.D.
WT	Poly (rAU+rAU), 40 bp	9.2 ± 10.6	103.2 ± 30.8	310 ± 340	91 ± 11

Supplementary Table 1. Summary of BLI analysis parameters. HC: heavy chain; LC: light chain. N.D.: not determined. K_d (kinetics) was calculated from k_{on}/k_{off} determined by kinetic analyses; K_d (steady state) was derived by curve-fitting sensor responses (nm) as functions of dsRNA concentrations. See Methods for details. Values are mean ± s.d. of three biologically independent samples. All J2 used in BLI analyses are in the form of IgG.

	J2 Fab bound to 23-bp dsRNA
**Data collection**	
Space group	$P\ 2\ 2_1\ 2_1$
Cell dimensions	
a, b, c (Å)	65.391 92.374 194.663
α, β, γ (°)	90, 90, 90
Resolution (Å)	54.28 – 2.85 (2.90 – 2.85)
R_{sym} or R_{merge}	0.452 (2.77)
R_{pim}	0.085 (0.54)
I / σI	7.3 (1.0)
$CC_{1/2}$	0.992 (0.444)
Completeness (%)	100.0 (98.1)
Redundancy	29.2 (26.6)
**Refinement**	
Resolution (Å)	20.64 – 2.85 (2.96 – 2.85)
No. reflections	28092 (3013)
R_{work} / R_{free}	0.212/0.262 (0.297/0.369)
No. atoms	7309
Macromolecule	7234
Water	26
Ligand	49
B-factors	50.82
Macromolecule	50.84
Water	36.61
Ligands	55.69
R.m.s. deviations	
Bond lengths (Å)	0.002
Bond angles (°)	0.52
Maximum-likelihood coordinate precision (Å)	0.36
Protein Data Bank (PDB) accession code	

Supplementary Table 2. Summary of data collection and refinement statistics for the co-crystal structures of J2 Fab bound to a 23-bp dsRNA.

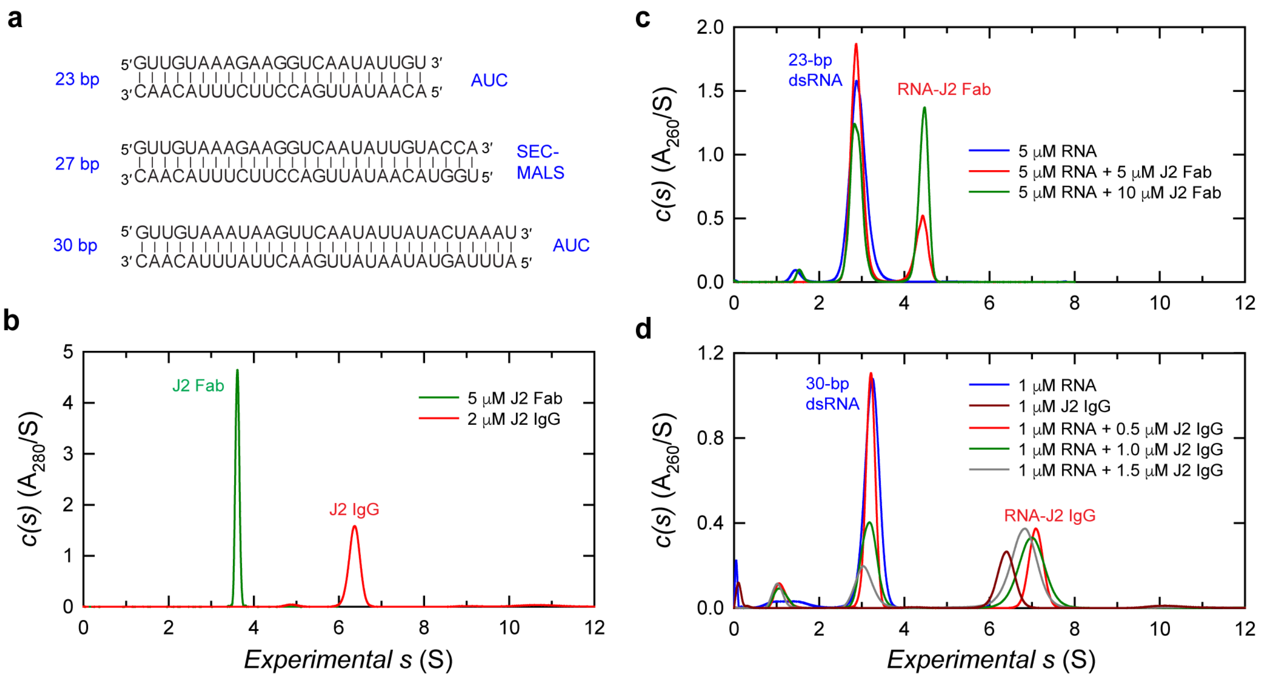

Supplementary Figure 1. Sedimentation Velocity – Analytical Ultracentrifugation (SV-AUC) analyses of dsRNA interactions with J2 Fab and J2 IgG. a. Sequences of dsRNAs used for AUC and SEC-MALS analyses. **b**. Absorbance c(s) distributions of 5 μM J2 Fab (green) and 2 μM J2 IgG (red). The sample of J2 Fab shows a single species at 3.61 S and 52 kDa indicative of a monomeric Fab. The sample of J2 IgG shows a species at 6.35 S and 147 kDa, indicative of a monomeric IgG. The IgG monomer accounts for 81% of the absorbing sedimenting signal; the dimer (~ 7%) and faster sedimenting material contribute to the signal. **c**. Titration of a 23-bp dsRNA (**a**) with J2 Fab (**b**). The 23-bp dsRNA shows a species at 2.90 S and 14 kDa, indicative of the expected dsRNA duplex. Adding of 1.0 (red) or 2.0 (green) molar equivalents of J2 Fab results in the contribution of a species at 4.39 S and 52 kDa. This species sediments faster than the J2 Fab (3.61 S), indicating a 1:1 RNA:Fab complex. **d**. Titration of a 30-bp dsRNA (17% GC, **a**) with J2 IgG (**b**). The 30-bp dsRNA shows a species at 3.23 S and 19 kDa, indicative of the expected dsRNA duplex. Adding 0.5 equivalents of J2 IgG (red) results in the contribution of a species at 7.09 S and 142 kDa. The species sediments faster than J2 IgG (6.35 S), indicating a 1:1 RNA:IgG complex. The addition of 1.0 (green) or 1.5 (gray) molar equivalents of J2 IgG shows a similar faster sedimenting species with increasing contributions from unresolved free IgG. The plot in brown shows a portion of the c(s) sedimentation profile for 1 μM free J2 IgG. Sample losses were observed due to the formation of large aggregates between the dsRNA and the IgG multimer and faster sedimenting material. Data for (**b**) were collected at 50,000 rpm and 280 nm, whereas data for (**c**) and (**d**) were collected at 50,000 rpm and 260 nm. 12 mm pathlength cells were used in (**b**) and (**d**), whereas 3 mm pathlength cells were used in (**c**).

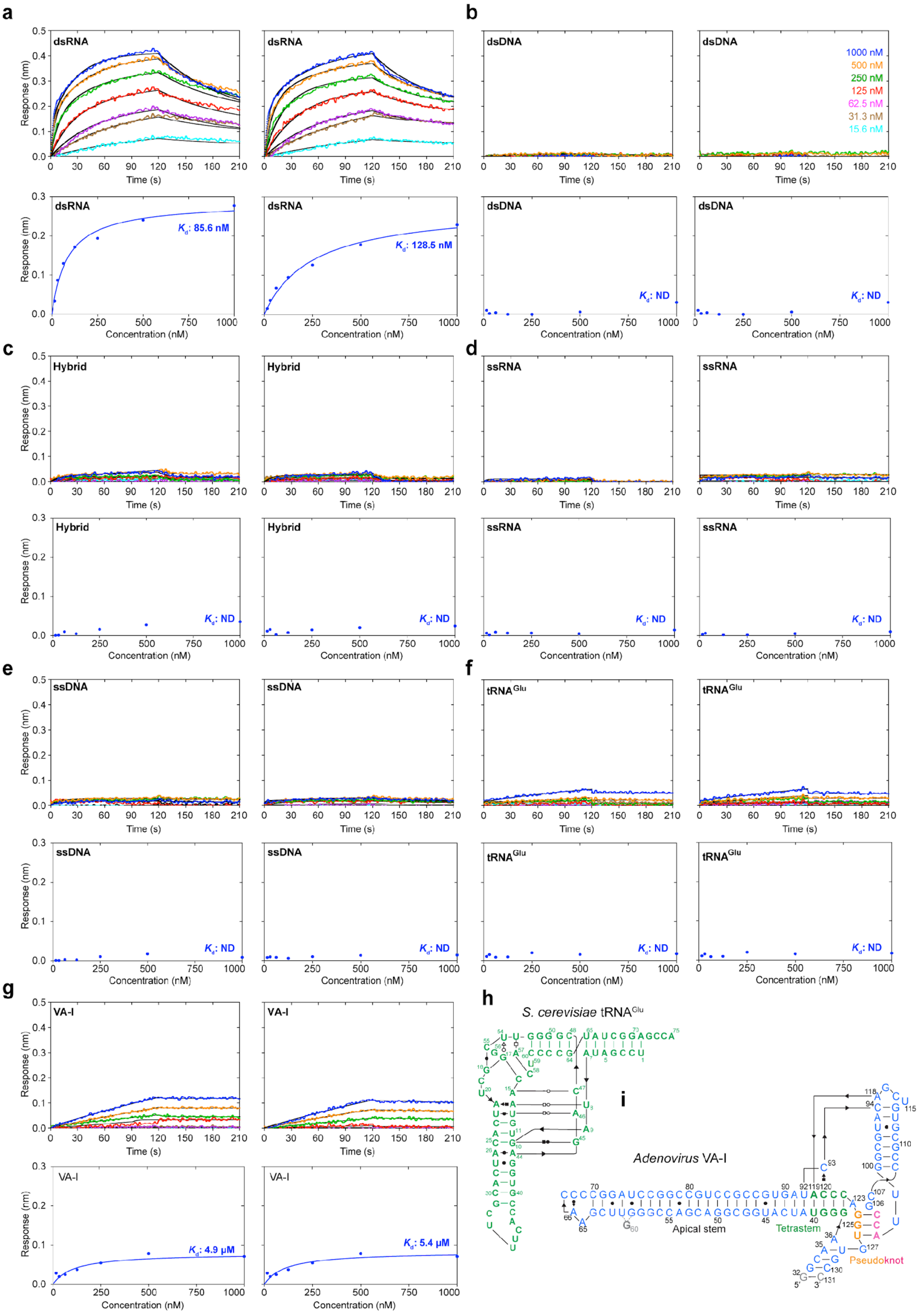
a
b
c
d
e
f
g
h
i
dsRNA
dsDNA
Hybrid
ssRNA
ssDNA
tRNA^Glu
VA-I
Response (nm)
Time (s)
Concentration (nM)
1000 nM
500 nM
250 nM
125 nM
62.5 nM
31.3 nM
15.6 nM
Kd: 85.6 nM
Kd: 128.5 nM
Kd: ND
Kd: 4.9 μM
Kd: 5.4 μM
S. cerevisiae tRNA^Glu
Adenovirus VA-I
Apical stem
Tetrastem
Pseudoknot

Supplementary Figure 2. J2 IgG binding to different types of nucleic acids. a-**g**, Additional BLI sensorgrams of J2 IgG binding to dsRNA (**a**), dsDNA (**b**), RNA-DNA hybrid (**c**), ssRNA (**d**), ssDNA (**e**), $tRNA^{Glu}$ (**f**), or VA-I (**g**) at 400 mM KCl. **h**. Secondary structure of $tRNA^{Glu}$ used in **f**. **i**. Secondary structure of VA-I used in **g**. Other nucleic acids used in (**a**-**f**) are shown in Fig. 1a.

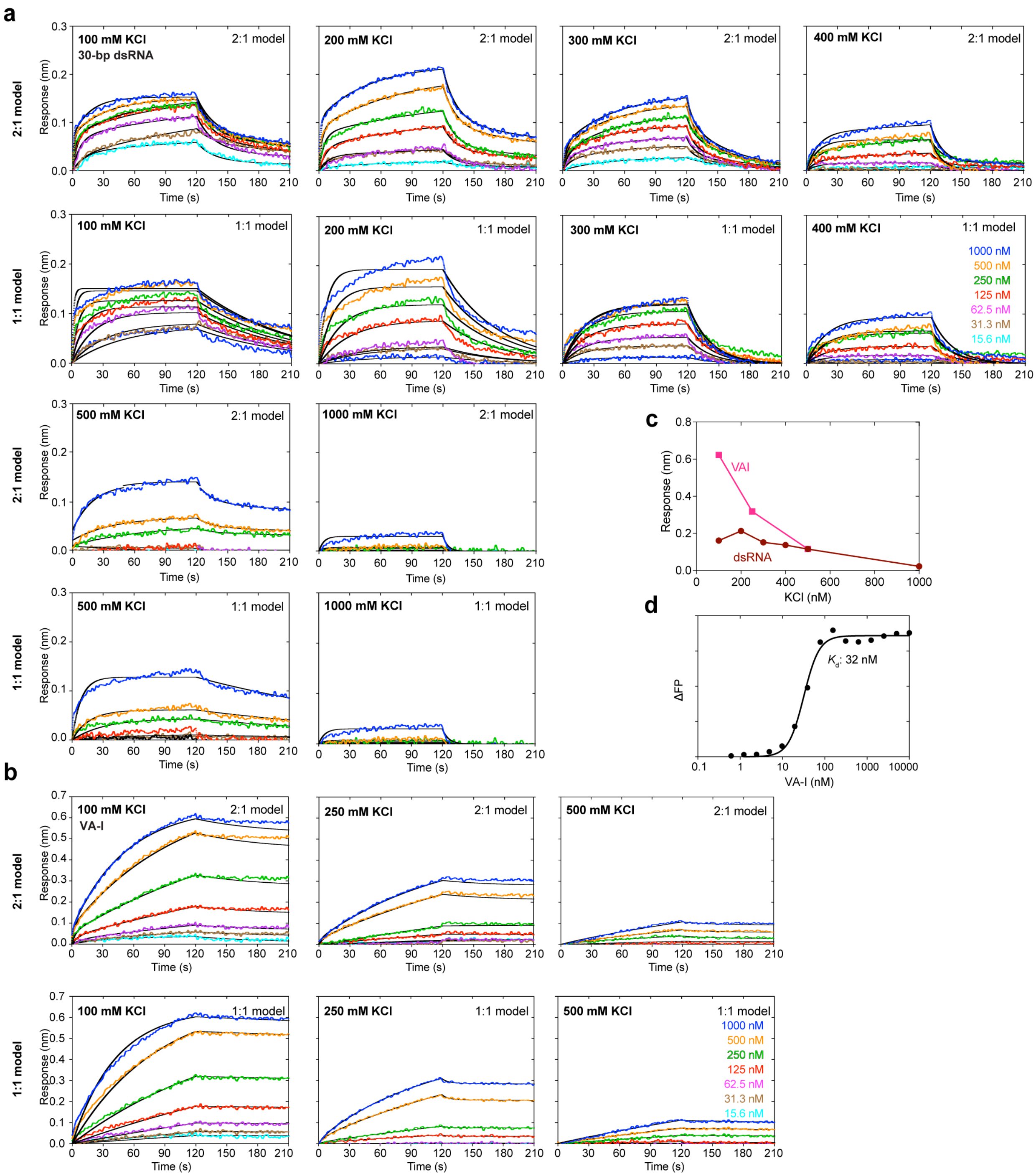

Supplementary Figure 3. Effects of ionic strengths on J2 IgG binding to dsRNA or VA-I. a, BLI sensorgrams of J2 binding to a 30-bp dsRNA, at 100, 200, 300, 400, 500, or 1000 mM KCl, fit to the 2:1 model (heterogeneous ligand, upper), or 1:1 model (lower). **b**. BLI sensorgrams of J2 binding to VA-I RNA at 100, 250, or 500 mM KCl, fit to the 2:1 model (upper), or 1:1 model (lower). **c**, Plots of maximum sensor responses as functions of KCl concentrations, derived from **a** & **b**. **d**, Additional bimolecular fluorescence polarization (FP) analysis of J2 IgG binding to a 3′-FAM-labelled VA-I RNA. ΔFP: change in FP expressed in dimensionless millipolarization (mP) units.

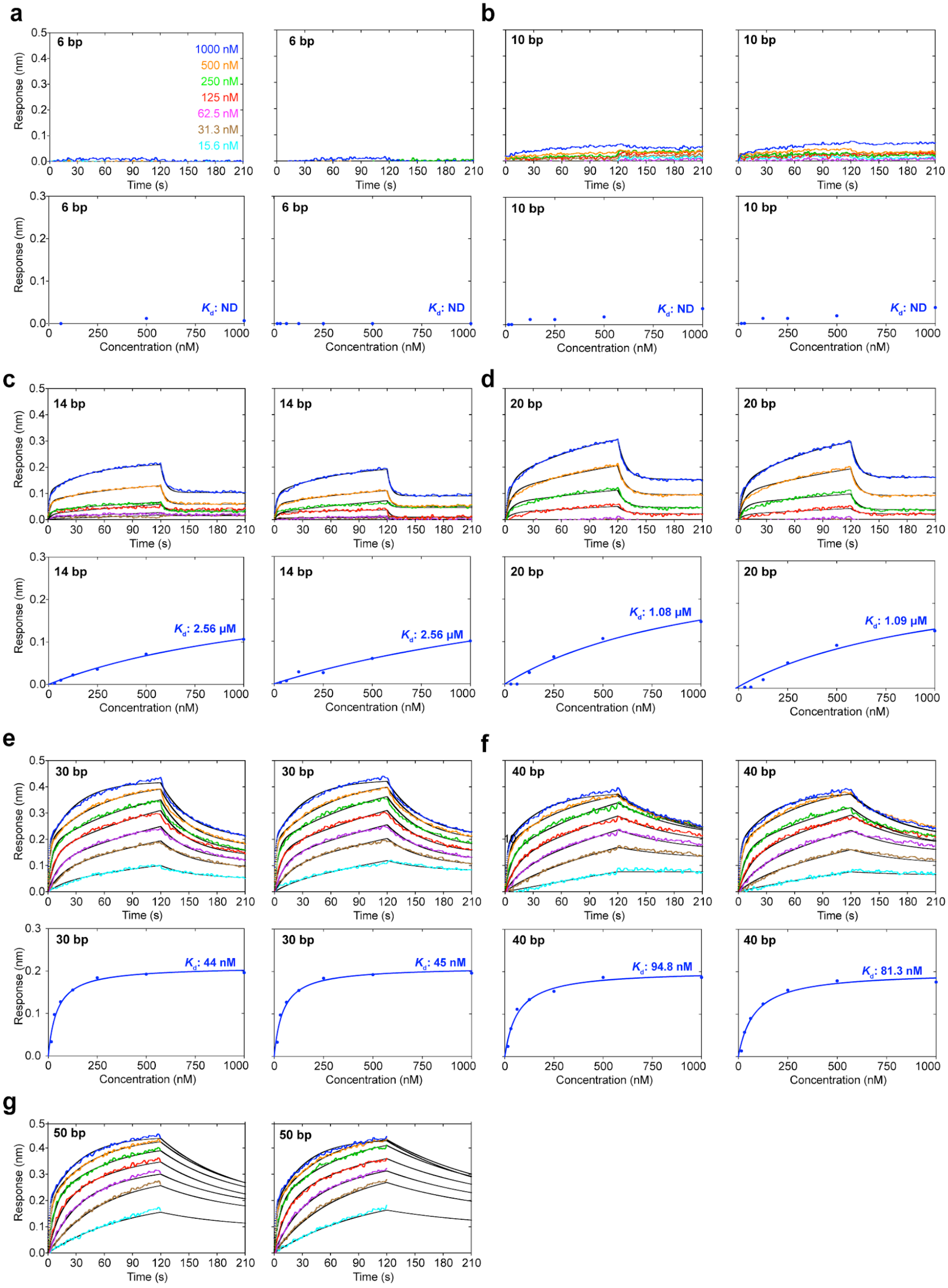

a
b
c
d
e
f
g
6 bp
10 bp
14 bp
20 bp
30 bp
40 bp
50 bp
1000 nM
500 nM
250 nM
125 nM
62.5 nM
31.3 nM
15.6 nM
Response (nm)
Time (s)
Concentration (nM)
Kd: ND
Kd: 2.56 μM
Kd: 1.08 μM
Kd: 1.09 μM
Kd: 44 nM
Kd: 45 nM
Kd: 94.8 nM
Kd: 81.3 nM

Supplementary Figure 4. J2 IgG binding to dsRNAs of different lengths. a-g, Additional BLI sensorgrams (upper) and steady-state analyses (lower) of J2 IgG binding to 6 (**a**), 10 (**b**), 14 (**c**), 20 (**d**), 30 (**e**), 40 (**f**), or 50 bp (**g**) dsRNAs. Sequences shown in Fig. 1e. Due to significant dependency of binding on ionic strength, 100 mM KCl was used for 6, 10, 14, and 20 bp dsRNAs; 200 mM KCl for 30 bp dsRNA; 400 mM KCl for 40 and 50 bp dsRNAs.

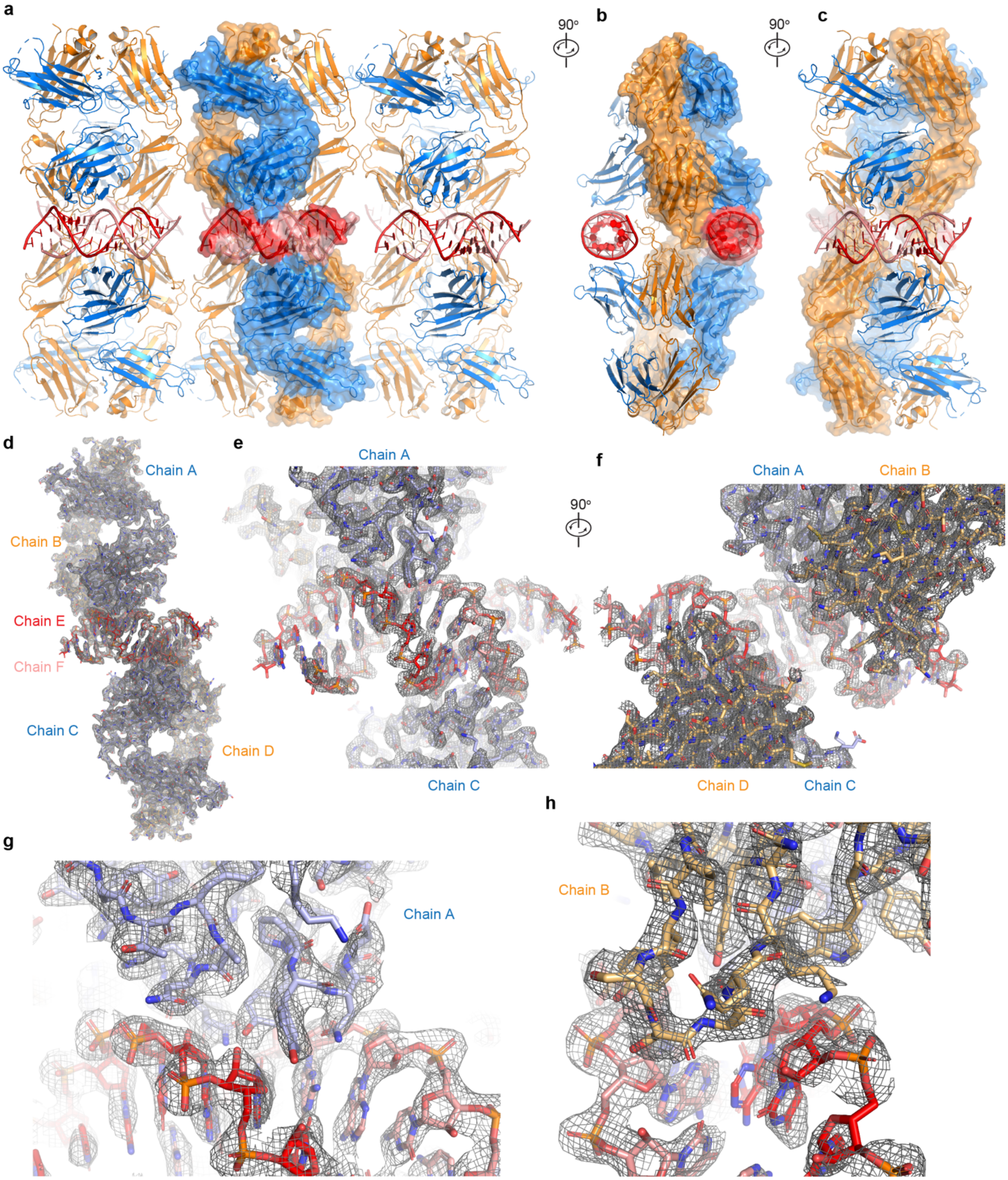

a
90°
b
90°
c
d
Chain A
Chain B
Chain E
Chain F
Chain C
Chain D
e
Chain A
Chain C
f
90°
Chain A
Chain B
Chain D
Chain C
g
Chain A
h
Chain B

Supplementary Figure 5. Crystal packing arrangements and representative electron densities. **a**, Crystal-packing arrangements of the J2 Fab – dsRNA (2:1) molecular assemblies. The heavy and light chains of the J2 Fabs are shown in blue and orange, respectively. The two dsRNA strands are in red and salmon. Contents within a single asymmetric unit are highlighted in translucent surface representation. **b**, 90° rotated view of **a**, showing two asymmetric units. **c**, 90° rotated view of **b**, showing two asymmetric units, and a rear view relative to **a**. **d**, Composite simulated anneal-omit 2Fo-Fc density calculated using the final model of two J2 Fabs bound to a 23-bp dsRNA, superimposed with the final refined model, contoured at 0.8 σ. **e**, **f**, Two views of a portion of the map in **d** showing the J2 interface with the dsRNA strands. **g**, Portion of the map in **d** showing the J2 heavy chain interface with the dsRNA. **h**, Portion of the map in **d** showing the J2 light chain interface with the dsRNA.

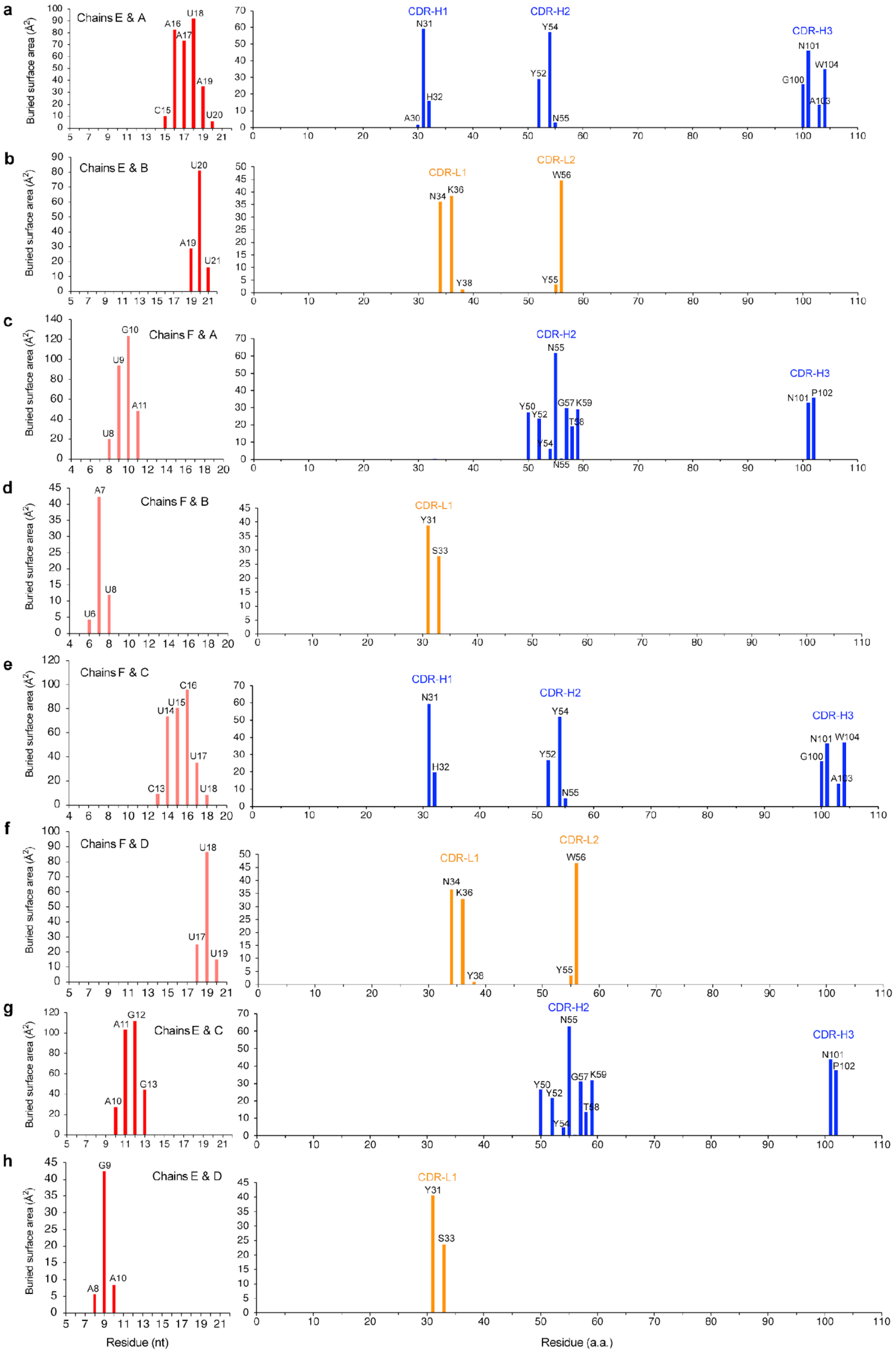

a
Chains E & A
CDR-H1
CDR-H2
CDR-H3
b
Chains E & B
CDR-L1
CDR-L2
c
Chains F & A
CDR-H2
CDR-H3
d
Chains F & B
CDR-L1
e
Chains F & C
CDR-H1
CDR-H2
CDR-H3
f
Chains F & D
CDR-L1
CDR-L2
g
Chains E & C
CDR-H2
CDR-H3
h
Chains E & D
CDR-L1
Buried surface area (Å²)
Residue (nt)
Residue (a.a.)

Supplementary Figure 6. Solvent-accessible surfaces buried at the J2 Fab – dsRNA interfaces. **a**-**d**, Plots of solvent-accessible surface area (Å^2) buried per residue on the dsRNA strands (left, chain E or F) and the first J2 Fab (right, heavy chain A in blue; light chain B in orange). **e**-**h**, Plots of solvent-accessible surface area (Å^2) buried per residue on the dsRNA strands (left, chain F or E) and the second J2 Fab (right, heavy chain C in blue; light chain D in orange).

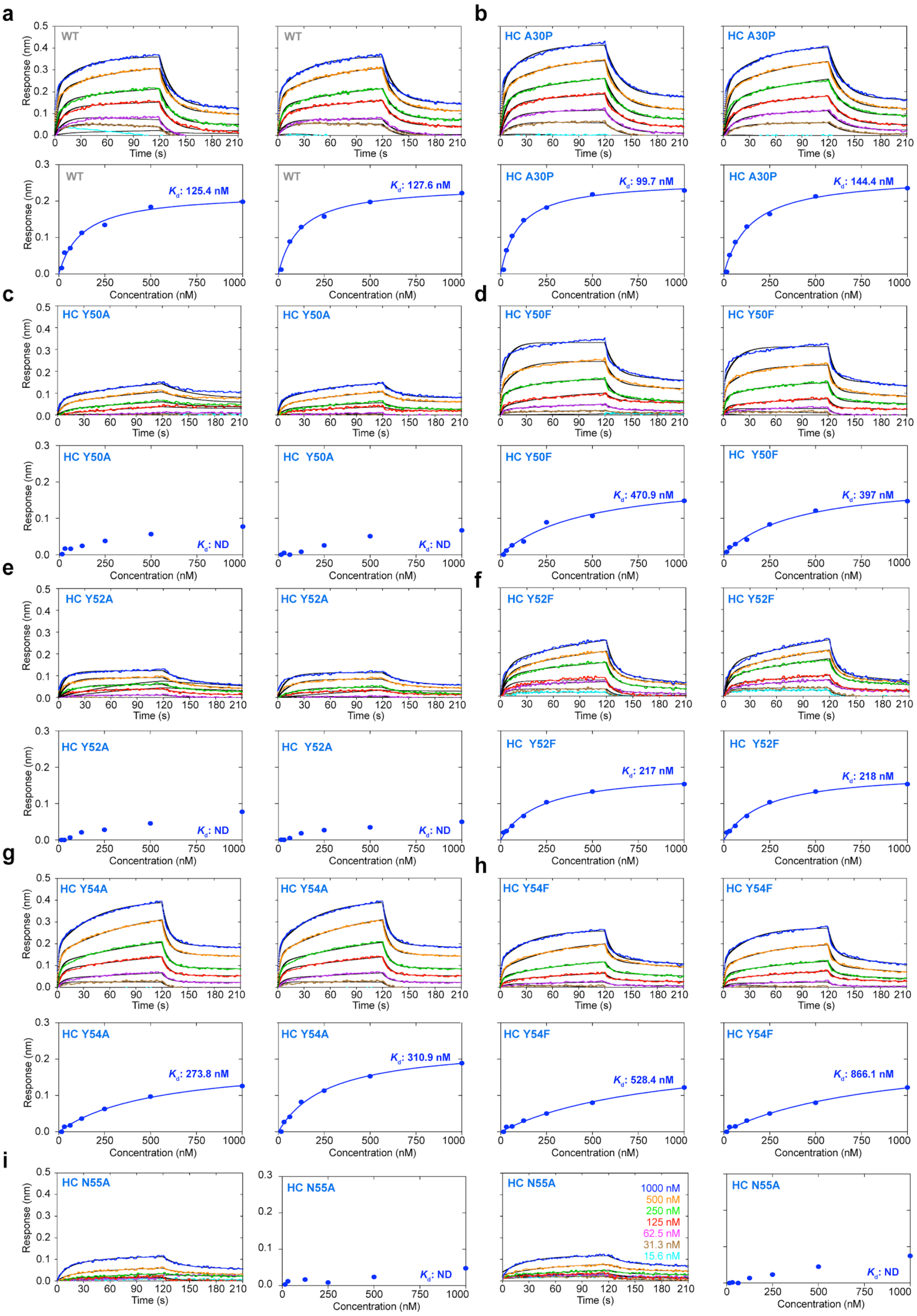

a
WT
Kd: 125.4 nM
Kd: 127.6 nM
b
HC A30P
Kd: 99.7 nM
Kd: 144.4 nM
c
HC Y50A
Kd: ND
d
HC Y50F
Kd: 470.9 nM
Kd: 397 nM
e
HC Y52A
Kd: ND
f
HC Y52F
Kd: 217 nM
Kd: 218 nM
g
HC Y54A
Kd: 273.8 nM
Kd: 310.9 nM
h
HC Y54F
Kd: 528.4 nM
Kd: 866.1 nM
i
HC N55A
Kd: ND
1000 nM
500 nM
250 nM
125 nM
62.5 nM
31.3 nM
15.6 nM
Response (nm)
Time (s)
Concentration (nM)

Supplementary Figure 7. Mutational analyses of the J2-dsRNA interface, part 1. a-i, Additional BLI sensorgrams (upper or left) and steady-state analyses (lower or right) of WT and mutant J2 IgG binding to a 20-bp dsRNA shown in Fig. 1e, at 100 mm KCl

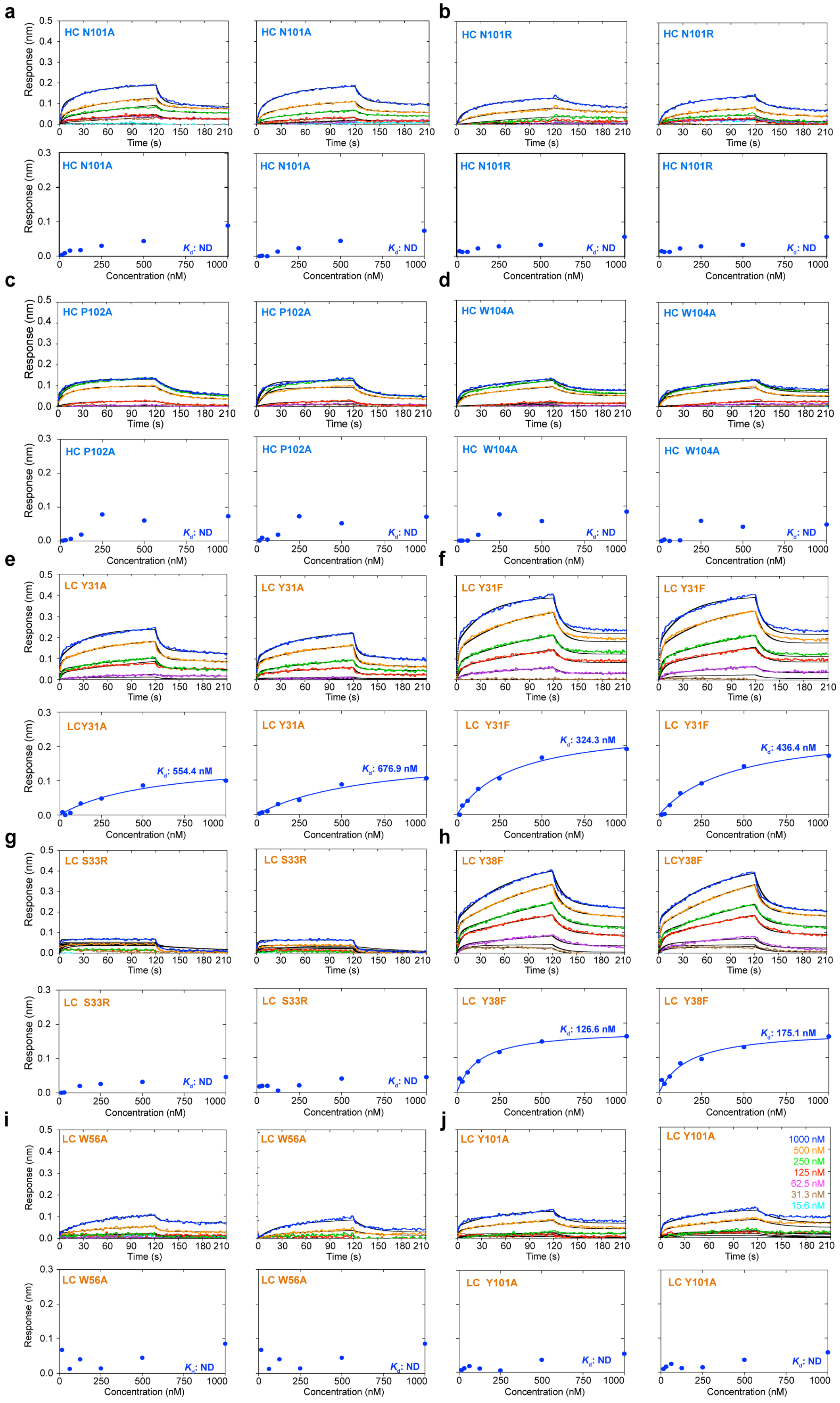

a
b
c
d
e
f
g
h
i
j
HC N101A
HC N101R
HC P102A
HC W104A
LC Y31A
LC Y31F
LC S33R
LC Y38F
LC W56A
LC Y101A
Response (nm)
Time (s)
Concentration (nM)
Kd: ND
Kd: 554.4 nM
Kd: 676.9 nM
Kd: 324.3 nM
Kd: 436.4 nM
Kd: 126.6 nM
Kd: 175.1 nM
1000 nM
500 nM
250 nM
125 nM
62.5 nM
31.3 nM
15.6 nM

Supplementary Figure 8. Mutational analyses of the J2-dsRNA interface, part 2. a-j, Additional BLI sensorgrams (upper) and steady-state analyses (lower) of WT and mutant J2 IgG binding to a 20-bp dsRNA shown in Fig. 1e, at 100 mm KCl.

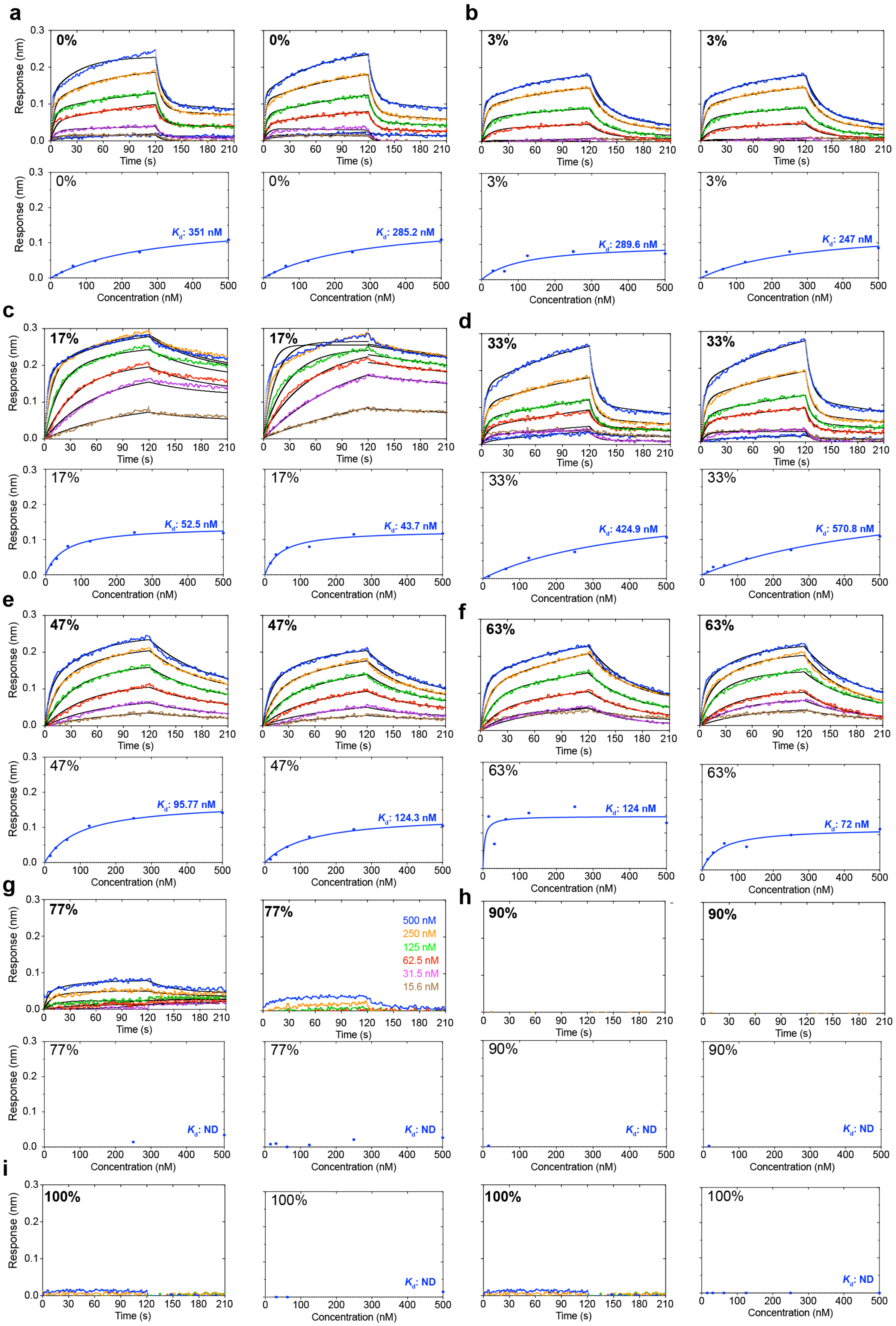
a
b
c
d
e
f
g
h
i
Response (nm)
Time (s)
Concentration (nM)
0%
3%
17%
33%
47%
63%
77%
90%
100%
Kd: 351 nM
Kd: 285.2 nM
Kd: 289.6 nM
Kd: 247 nM
Kd: 52.5 nM
Kd: 43.7 nM
Kd: 424.9 nM
Kd: 570.8 nM
Kd: 95.77 nM
Kd: 124.3 nM
Kd: 124 nM
Kd: 72 nM
Kd: ND
500 nM
250 nM
125 nM
62.5 nM
31.5 nM
15.6 nM

Supplementary Figure 9. Effects of GC contents on J2 IgG binding. a-i, Additional BLI sensorgrams (upper or left) and steady-state analyses (lower or right) of J2 binding to 30-bp dsRNAs shown in Fig. 4a, with GC contents of 0% (**a**), 3% (**b**), 17% (**c**), 33% (**d**), 47% (**e**), 63% (**f**), 77% (**g**), 90% (**h**), or 100% bp (**i**), at 200 mM KCl.

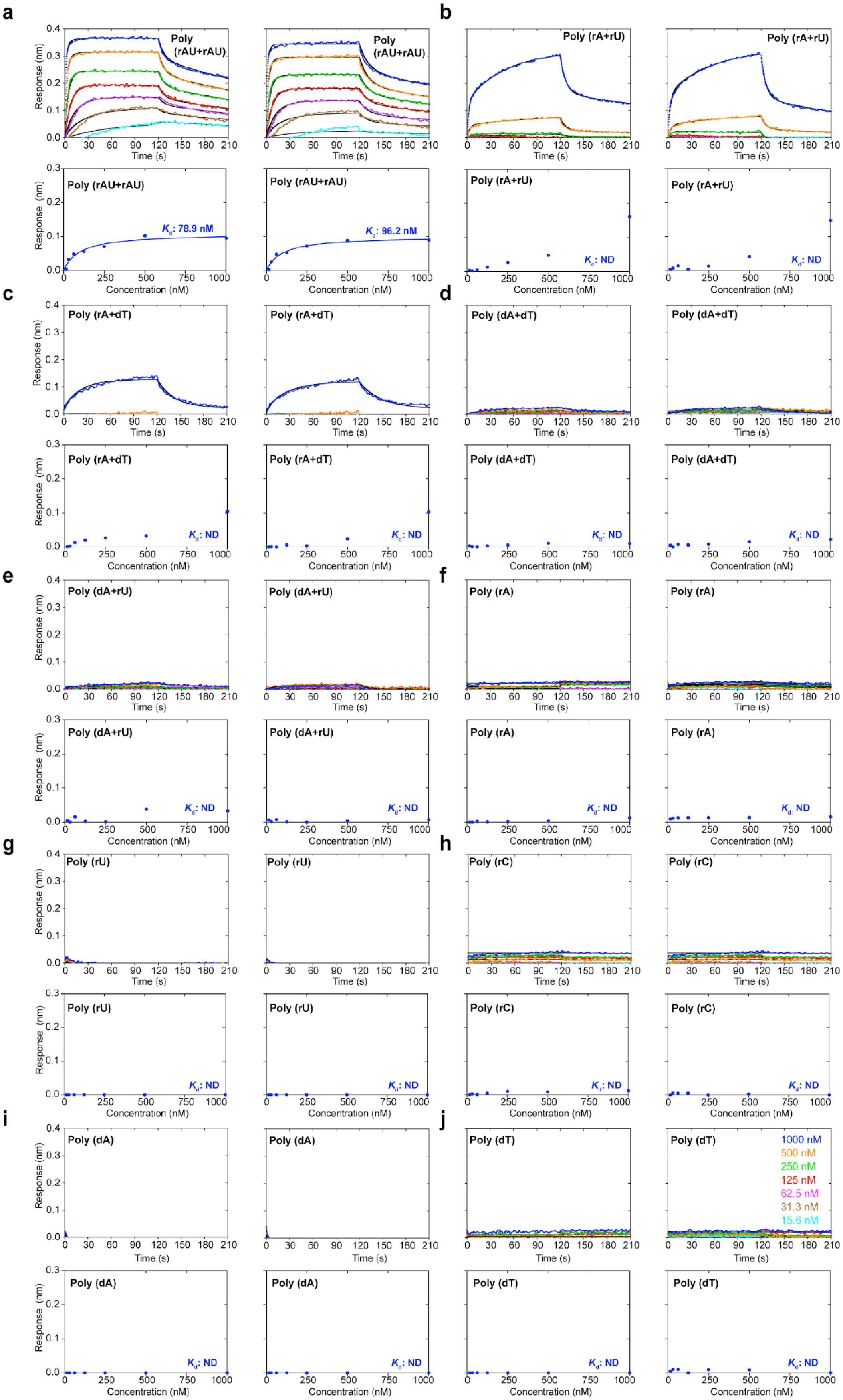
a
b
c
d
e
f
g
h
i
j
Poly (rAU+rAU)
Poly (rA+rU)
Poly (rA+dT)
Poly (dA+dT)
Poly (dA+rU)
Poly (rA)
Poly (rU)
Poly (rC)
Poly (dA)
Poly (dT)
K_d: 78.9 nM
K_d: 96.2 nM
K_d: ND
Response (nm)
Time (s)
Concentration (nM)
1000 nM
500 nM
250 nM
125 nM
62.5 nM
31.3 nM
15.6 nM

Supplementary Figure 10. J2 IgG interaction with homotypic repeat nucleic acid sequences. **a-j**, Additional BLI sensorgrams (upper) and steady-state analyses (lower) of J2 binding to repetitive nucleic acid sequences shown in Fig. 5a, at 400 mM KCl.
